# Supplementary figures and images for: Helicobacter pylori virulence factors: relationship between genetic variability and phylogeographic origin
Source: PeerJ. 2021 Nov 26;9:e12272. doi: 10.7717/peerj.12272 (PMC8628625; doi:10.7717/peerj.12272)

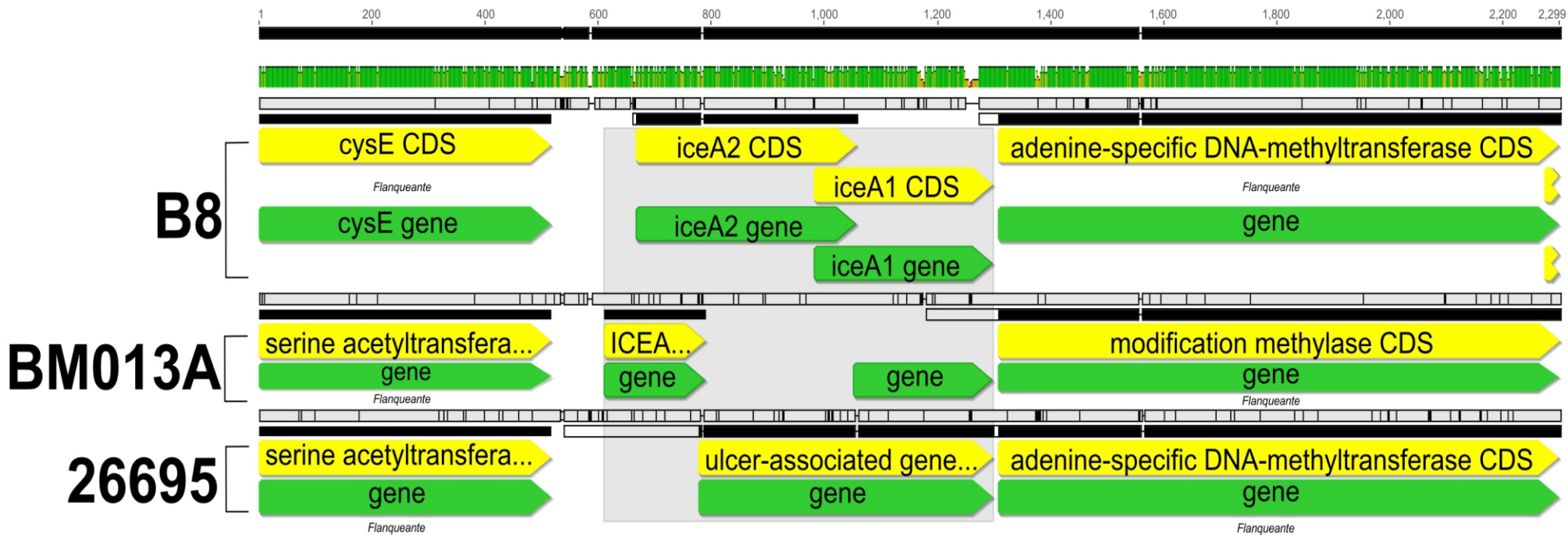

Supplement: Supplemental Information 1 — The orthologous regions were analyzed using Muscle alignment by Geneious program. Representative strains were used with iceA1 and iceA2 alleles (B8 and BM013A) and with a single copy of iceA (26695). [file peerj-09-12272-s001.pdf]
