# Supplementary material for: Helicobacter pylori virulence factors: relationship between genetic variability and phylogeographic origin
Source: PeerJ. 2021 Nov 26;9:e12272. doi: 10.7717/peerj.12272 (PMC8628625; doi:10.7717/peerj.12272)
Supplement: Supplemental Information 2 — Phylogenetic tree obtained using GRIMM program. All ancestral orders (A60 to A117) shown in each node in the phylogeny. The number preceded by the plus symbol (+) indicates the number of inversions in each branch. Colored circles show the phylogeographic origin and colored triangles show the pathogenic phenotype. [file peerj-09-12272-s002.pdf]

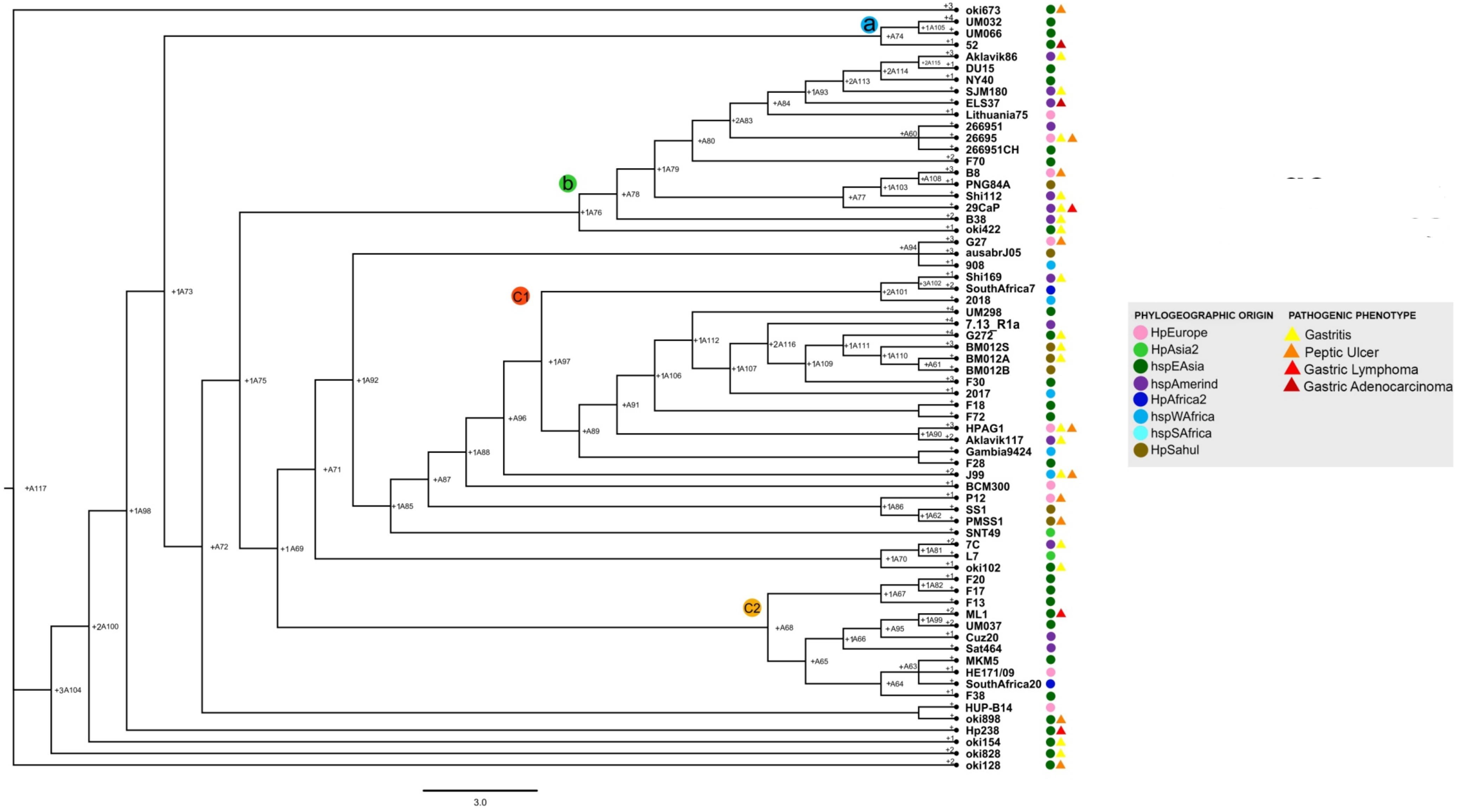
oki673
UM032
UM066
52
Aklavik86
DU15
NY40
SJM180
ELS37
Lithuania75
266951
26695
266951CH
F70
B8
PNG84A
Shi112
29CaP
B38
oki422
G27
ausabrJ05
908
Shi169
SouthAfrica7
2018
UM298
7.13_R1a
G272
BM012S
BM012A
BM012B
F30
2017
F18
F72
HPAG1
Aklavik117
Gambia9424
F28
J99
BCM300
P12
SS1
PMSS1
SNT49
7C
L7
oki102
F20
F17
F13
ML1
UM037
Cuz20
Sat464
MKM5
HE171/09
SouthAfrica20
F38
HUP-B14
oki898
Hp238
oki154
oki828
oki128
a
b
C1
C2
3.0
PHYLOGEOGRAPHIC ORIGIN
HpEurope
HpAsia2
hspEAsia
hspAmerind
HpAfrica2
hspWAfrica
hspSAfrica
HpSahul
PATHOGENIC PHENOTYPE
Gastritis
Peptic Ulcer
Gastric Lymphoma
Gastric Adenocarcinoma
