# Supplementary material for: Helicobacter pylori virulence factors: relationship between genetic variability and phylogeographic origin
Source: PeerJ. 2021 Nov 26;9:e12272. doi: 10.7717/peerj.12272 (PMC8628625; doi:10.7717/peerj.12272)
Supplement: Supplemental Information 3 [file peerj-09-12272-s003.docx]

Supplementary Table S1. *Helicobacter pylori* strains analyzed. ID, phylogeography origin and pathogenic phenotype by Strain are identified.

| Strain | ID number | Phylogeographic origin | Pathogenic phenotype |
| --- | --- | --- | --- |
| BM013A | NZ_CP007604 | HpSahul (Wattam et al., 2013) | ---- |
| BM012B | NZ_CP007605 | HpSahul (Wattam et al., 2013) | ---- |
| BM013B | NZ_CP007606 | HpSahul (Wattam et al., 2013) | ---- |
| Hp238 | NZ_CP010013 | hspEAsia (Wattam et al., 2013) | Gastric Lymphoma (Wattam et al., 2013) |
| 26695-1MET | NZ_CP010436 | hspAmerind (Wattam et al., 2013) | ---- |
| BM013A | NZ_CP007604 | HpSahul (Wattam et al., 2013) | ---- |
| BM012B | NZ_CP007605 | HpSahul (Wattam et al., 2013) | ---- |
| Hp238 | NZ_CP010013 | hspEAsia (Wattam et al., 2013) | Gastric Lymphoma (Wheeler et al., 2006) |
| 29CaP | NZ_CP012907 | hspAmerind (Wattam et al., 2013) | Gastritis/ Gastric Adenocarcinoma (Wattam et al., 2013) |
| 7C | NZ_CP012905 | hspAmerind (Wattam et al., 2013) | Gastritis (Wheeler et al., 2006) |
| L7 | NZ_CP011482 | HpAsia2 (Wattam et al., 2013) | ---- |
| DU15 | NZ_CP011483 | hspEAsia (Wattam et al., 2013) | ---- |
| CC33C | NZ_CP011484 | hspSAfrica (Wattam et al., 2013) | ---- |
| ausabrJ05 | NZ_CP011485 | HpSahul (Wattam et al., 2013) | ---- |
| K26A1 | NZ_CP011486 | hspSAfrica (Wattam et al., 2013) | ---- |
| PNG84A | NZ_CP011487 | HpSahul (Wattam et al., 2013) | ---- |
| G272 | NZ_CP022409 | hspEAsia (Wattam et al., 2013) | Gastritis (Wattam et al., 2013) |
| HPJP26 | NZ_CP023448 | hspAmerind (Wattam et al., 2013) | ---- |
| dRdM1 | CP026325 | hspAmerind (Wattam et al., 2013) | ---- |
| 26695-dRdM1dM2 | CP026323 | hspAmerind (Wattam et al., 2013) | ---- |
| 26695-dR | CP026326 | hspAmerind (Wattam et al., 2013) | ---- |
| 26695-dRdM2 | CP026324 | hspAmerind (Wattam et al., 2013) | ---- |
| 7.13_R1c | CP024073 | hspAmerind (Wattam et al., 2013) | ---- |
| 7.13_R3a | CP024077 | hspAmerind (Wattam et al., 2013) | ---- |
| 7.13_R2b | CP024075 | hspAmerind (Wattam et al., 2013) | ---- |
| 7.13_R1a | CP024071 | hspAmerind (Wattam et al., 2013) | ---- |
| dRdM2addM2 | CP026515 | hspAmerind (Wheeler et al., 2006) | ---- |
| HE171/09 | LT635474 | HpEurope (Wheeler et al., 2006) | ---- |
| HE143/09 | LT635458 | HpEurope (Wheeler et al., 2006) | ---- |
| HE178/09 | LT635460 | HpEurope (Wheeler et al., 2006) | ---- |
| HE132/09 | LT635459 | HpEurope (Wheeler et al., 2006) | ---- |
| HE134/09 | LT635476 | HpEurope (Wheeler et al., 2006) | ---- |
| HE141/09 | LT635471 | HpEurope (Wheeler et al., 2006) | ---- |
| HE136/09 | LT635473 | HpEurope (Wheeler et al., 2006) | ---- |
| HE101/09 | LT635456 | HpEurope (Wheeler et al., 2006) | ---- |
| HE142/09 | LT635478 | HpEurope (Wheeler et al., 2006) | ---- |
| HE170/09 | LT635472 | HpEurope (Wheeler et al., 2006) | ---- |
| HE147/09 | LT635477 | HpEurope (Wheeler et al., 2006) | ---- |
| BCM-300 | LT837687 | HpEurope (Wheeler et al., 2006) | ---- |
| HE93/10_v1 | LT838273 | HpEurope (Wheeler et al., 2006) | ---- |
| MKM5 | NZ_AP017360 | hspEAsia (Wattam et al., 2013) | ---- |
| F28 | NZ_AP017339 | hspEAsia (Wattam et al., 2013) | ---- |
| F38 | NZ_AP017341 | hspEAsia (Wattam et al., 2013) | ---- |
| F63 | NZ_AP017346 | hspEAsia (Wattam et al., 2013) | ---- |
| F51 | NZ_AP017343 | hspEAsia (Wattam et al., 2013) | ---- |
| F78 | NZ_AP017352 | hspEAsia (Wattam et al., 2013) | ---- |
| F13 | NZ_AP017329 | hspEAsia (Wattam et al., 2013) | ---- |
| F17 | NZ_AP017330 | hspEAsia (Wattam et al., 2013) | ---- |
| F18 | NZ_AP017331 | hspEAsia (Wattam et al., 2013) | ---- |
| F209 | NZ_AP017332 | hspEAsia (Wattam et al., 2013) | ---- |
| F20 | NZ_AP017333 | hspEAsia (Wattam et al., 2013) | ---- |
| F210 | NZ_AP017334 | hspEAsia (Wattam et al., 2013) | ---- |
| F211 | NZ_AP017335 | hspEAsia (Wattam et al., 2013) | ---- |
| F21 | NZ_AP017336 | hspEAsia (Wattam et al., 2013) | ---- |
| F23 | NZ_AP017337 | hspEAsia (Wattam et al., 2013) | ---- |
| F24 | NZ_AP017338 | hspEAsia (Wattam et al., 2013) | ---- |
| F55 | NZ_AP017345 | hspEAsia (Wattam et al., 2013) | ---- |
| F67 | NZ_AP017348 | hspEAsia (Wattam et al., 2013) | ---- |
| F70 | NZ_AP017349 | hspEAsia (Wattam et al., 2013) | ---- |
| F72 | NZ_AP017350 | hspEAsia (Wattam et al., 2013) | ---- |
| F75 | NZ_AP017351 | hspEAsia (Wattam et al., 2013) | ---- |
| F90 | NZ_AP017354 | hspEAsia (Wattam et al., 2013) | ---- |
| F94 | NZ_AP017355 | hspEAsia (Wattam et al., 2013) | ---- |
| MKF10 | NZ_AP017356 | hspEAsia (Wheeler et al., 2006) | ---- |
| MKF3 | NZ_AP017357 | hspEAsia (Wheeler et al., 2006) | ---- |
| MKF8 | NZ_AP017358 | hspEAsia (Wheeler et al., 2006) | ---- |
| MKM1 | NZ_AP017359 | hspEAsia (Wheeler et al., 2006) | ---- |
| MKM6 | NZ_AP017362 | hspEAsia (Wheeler et al., 2006) | ---- |
| ML1 | AP014710 | hspEAsia (Wheeler et al., 2006) | Gastric Lymphoma (Wattam et al., 2013) |
| ML2 | AP014711 | hspEAsia (Wheeler et al., 2006) | Gastric Lymphoma (Wattam et al., 2013) |
| ML3 | AP014712 | hspEAsia (Wheeler et al., 2006) | Gastric Lymphoma (Wattam et al., 2013) |
| 2017 | NC_017374 | hspWAfrica (Kumar et al., 2015) | ---- |
| 2018 | NC_017381 | hspWAfrica (Kumar et al., 2015) | ---- |
| 26695 | NC_000915 | HpEurope (Kumar et al., 2015) | Gastritis/ Peptic Ulcer (Wattam et al., 2013) |
| 26695-1 | NZ_CP010435 | hspAmerind (Wheeler et al., 2006) | ---- |
| 26695-1CH | NZ_AP013355 | hspEAsia (Wheeler et al., 2006) | ---- |
| 26695-1CL | NZ_AP013356 | hspEAsia (Wheeler et al., 2006) | ---- |
| 35A | NC_017360 | hspEAsia (Kumar et al., 2015) | Gastritis/ Peptic Ulcer (Wheeler et al., 2006) |
| 51 | NC_017382 | hspEAsia (Kumar et al., 2015) | Gastritis/ Peptic Ulcer (Wheeler et al., 2006) |
| 52 | NC_017354 | hspEAsia (Kumar et al., 2015) | Gastric Adenocarcinoma (Wattam et al., 2013) |
| 83 | NC_017375 | hspEAsia (Kumar et al., 2015) | Gastritis/ Peptic Ulcer (Wheeler et al., 2006) |
| 908 | NC_017357 | hspWAfrica (Kumar et al., 2015) | ---- |
| Aklavik117 | NC_019560 | hspAmerind (Wheeler et al., 2006) | Gastritis (Wheeler et al., 2006) |
| Aklavik86 | NC_019563 | hspAmerind (Wheeler et al., 2006) | Gastritis (Wheeler et al., 2006) |
| B38 | NC_012973 | HpEurope (Wheeler et al., 2006) | Peptic Ulcer (Wheeler et al., 2006) |
| B8 | NC_014256 | HpEurope (Kumar et al., 2015) | Peptic Ulcer (Wheeler et al., 2006) |
| BM012A | NC_022886 | HpSahul (Wattam et al., 2013) | Gastritis (Wheeler et al., 2006) |
| BM012S | NC_022911 | HpSahul (Wattam et al., 2013) | Gastritis (Wheeler et al., 2006) |
| Cuz20 | NC_017358 | hspAmerind (Kumar et al., 2015) | ---- |
| ELS37 | NC_017063 | hspAmerind (Wheeler et al., 2006) | Gastric Adenocarcinoma (Wheeler et al., 2006) |
| F16 | NC_017368 | hspEAsia (Kumar et al., 2015) | ---- |
| F30 | NC_017365 | hspEAsia (Kumar et al., 2015) | ---- |
| F32 | NC_017366 | hspEAsia (Kumar et al., 2015) | ---- |
| F57 | NC_017367 | hspEAsia (Kumar et al., 2015) | ---- |
| G27 | NC_011333 | HpEurope (Kumar et al., 2015) | Peptic Ulcer (Wheeler et al., 2006) |
| Gambia94/24 | NC_017371 | hspWAfrica (Wattam et al., 2013) | ---- |
| HPAG1 | NC_008086 | HpEurope (Kumar et al., 2015) | Gastritis/ Peptic Ulcer (Wattam et al., 2013) |
| HUP-B14 | NC_017733 | HpEurope (Kumar et al., 2015) | ---- |
| India7 | NC_017372 | HpAsia2 (Wheeler et al., 2006) | ---- |
| J166 | NZ_CP007603 | HpEurope (Wheeler et al., 2006) | ---- |
| J99 | NC_000921 | hspWAfrica (Wheeler et al., 2006) | Gastritis/ Peptic Ulcer (Wheeler et al., 2006) |
| Lithuania75 | NC_017362 | HpEurope (Kumar et al., 2015) | ---- |
| NY40 | NZ_AP014523 | hspEAsia (Wattam et al., 2013) | ---- |
| OK113 | NC_020508 | hspEAsia (Kumar et al., 2015) | ---- |
| OK310 | NC_020509 | hspEAsia (Kumar et al., 2015) | ---- |
| P12 | NC_011498 | HpEurope (Kumar et al., 2015) | Peptic Ulcer (Wheeler et al., 2006) |
| PMSS1 | NZ_CP018823 | HpSahul (Wheeler et al., 2006) | Peptic Ulcer (Wattam et al., 2013) |
| PeCan18 | NC_017742 | hspAmerind (Wattam et al., 2013) | Gastric Adenocarcinoma (Wheeler et al., 2006) |
| PeCan4 | NC_014555 | hspAmerind (Kumar et al., 2015) | Gastric Adenocarcinoma (Wheeler et al., 2006) |
| Puno120 | NC_017378 | hspAmerind (Kumar et al., 2015) | Gastritis (Wheeler et al., 2006) |
| Puno135 | NC_017379 | hspAmerind (Kumar et al., 2015) | Gastritis (Wheeler et al., 2006) |
| Rif1 | NC_018937 | HpEurope (Kumar et al., 2015) | ---- |
| Rif2 | NC_018938 | HpEurope (Kumar et al., 2015) | ---- |
| SJM180 | NC_014560 | hspAmerind (Wattam et al., 2013) | Gastritis (Wheeler et al., 2006) |
| SNT49 | NC_017376 | HpAsia2 (Kumar et al., 2015) | ---- |
| SS1 | NZ_CP009259 | HpSahul (Wattam et al., 2013) | ---- |
| Sat464 | NC_017359 | hspAmerind (Kumar et al., 2015) | ---- |
| Shi112 | NC_017741 | hspAmerind (Kumar et al., 2015) | Gastritis (Wheeler et al., 2006) |
| Shi169 | NC_017740 | hspAmerind (Kumar et al., 2015) | Gastritis (Wheeler et al., 2006) |
| Shi417 | NC_017739 | hspAmerind (Kumar et al., 2015) | Gastritis (Wheeler et al., 2006) |
| Shi470 | NC_010698 | hspAmerind (Kumar et al., 2015) | Gastritis/ Peptic Ulcer (Wattam et al., 2013) |
| SouthAfrica20 | CP006691 | HpAfrica2 (Duncan et al., 2013) | ---- |
| SouthAfrica7 | NC_017361 | HpAfrica2 (Duncan et al., 2013) | ---- |
| UM032 | NC_021215 | hspEAsia (Wheeler et al., 2006) | ---- |
| UM037 | NC_021217 | hspEAsia (Wattam et al., 2013) | ---- |
| UM066 | NC_021218 | hspEAsia (Wheeler et al., 2006) | ---- |
| UM298 | NC_021882 | hspEAsia (Wheeler et al., 2006) | ---- |
| UM299 | NC_021216 | hspEAsia (Wheeler et al., 2006) | ---- |
| XZ274 | CP003419 | hspEAsia (Kumar et al., 2015) | Gastric Adenocarcinoma (Wheeler et al., 2006) |
| oki102 | NZ_CP006820 | hspEAsia (Wheeler et al., 2006) | Gastritis/ Atrophic Gastritis (Wheeler et al., 2006) |
| oki112 | NZ_CP006821 | hspEAsia (Wheeler et al., 2006) | Gastritis/ Atrophic Gastritis (Wheeler et al., 2006) |
| oki128 | NZ_CP006822 | hspEAsia (Wheeler et al., 2006) | Gastritis/ Atrophic Gastritis (Wheeler et al., 2006) |
| oki154 | NZ_CP006823 | hspEAsia (Wheeler et al., 2006) | Gastritis/ Atrophic Gastritis (Wheeler et al., 2006) |
| oki422 | NZ_CP006824 | hspEAsia (Wheeler et al., 2006) | Gastritis/ Atrophic Gastritis (Wheeler et al., 2006) |
| oki673 | NZ_CP006825 | hspEAsia (Wheeler et al., 2006) | Peptic Ulcer (Wheeler et al., 2006) |
| oki828 | NZ_CP006826 | hspEAsia (Wheeler et al., 2006) | Peptic Ulcer (Wheeler et al., 2006) |
| oki898 | NZ_CP006827 | hspEAsia (Wheeler et al., 2006) | Peptic Ulcer (Wheeler et al., 2006) |
| v225d | NC_017355 | hspAmerind (Kumar et al., 2015) | Gastritis (Wheeler et al., 2006) |

Bibliografía

Duncan, S. S., Bertoli, M. T., Kersulyte, D., Valk, P. L., Tamma, S., Segal, I., McClain, M. S., Cover, T. L., & Berg, D. E. (2013). Genome Sequences of Three hpAfrica2 Strains of Helicobacter pylori. Genome announcements, 1(5), e00729-13. <https://doi.org/10.1128/genomeA.00729-13>.

Kumar, N., Mariappan, V., Baddam, R., Lankapalli, A. K., Shaik, S., Goh, K. L., & Ahmed, N. (2015). Comparative genomic analysis of Helicobacter pylori from Malaysia identifies three distinct lineages suggestive of differential evolution. Nucleic acids research, 43(1), 324-335.

Wattam, A. R., Abraham, D., Dalay, O., Disz, T. L., Driscoll, T., Gabbard, J. L., & Machi, D. (2013). PATRIC, the bacterial bioinformatics database and analysis resource. Nucleic acids research, 42(D1), D581-D591. Recuperado en <https://www.patricbrc.org/view/GenomeList/?and(keyword(Helicobacter),keyword(pylori))#view_tab=genomes>

Wheeler, D. L., Barrett, T., Benson, D. A., Bryant, S. H., Canese, K., Chetvernin, V., & Geer, L. Y. (2006). Database resources of the national center for biotechnology information. Nucleic acids research, 35(suppl_1), D5-D12.
