# Supplementary material for: Helicobacter pylori virulence factors: relationship between genetic variability and phylogeographic origin
Source: PeerJ. 2021 Nov 26;9:e12272. doi: 10.7717/peerj.12272 (PMC8628625; doi:10.7717/peerj.12272)
Supplement: Supplemental Information 6 [file peerj-09-12272-s006.docx]

**Supplementary Table S4.** The list of strains grouped by the 66 different types of rearrangements in the adhesins genes.

| ***Unic Phylogeographical origin*** | ***Reference strain*** | | ***strains with similar rearrangement*** |
| --- | --- | --- | --- |
|  | |  | Rif1 (HpEurope) |
|  | |  | Rif2 (HpEurope) |
|  | |  | 26695-1MET (hspAmerind) |
|  | | 26695 | dRdM1 (hspAmerind)  26695-dRdM1dM2 (hspAmerind) |
|  | |  | 26695-dR (hspAmerind) |
|  | |  | 26695-dRdM2 (hspAmerind) |
|  | |  | dRdM2addM2 (hspAmerind) |
|  | |  | HE143/09 |
|  | |  | HE178/09 |
|  | |  | HE132/09 |
|  | |  | HE134/09 |
|  | |  | HE141/09 |
| **HpEurope** | | HE171/09 | HE136/09 |
|  | |  | HE101/09 |
|  | |  | HE142/09 |
|  | |  | HE170/09 |
|  | |  | HE147/09 |
|  | |  | HE93/10_v1 |
|  | | BCM-300 | ---- |
|  | | B38 | ---- |
|  | | B8 | ---- |
|  | |  | XZ274 (hspEAsia) |
|  | |  | 35A (hspEAsia) |
|  | | HUP-B14 | HPJP26 (hspAmerind) |
|  | |  | 83 (hspEAsia) |
|  | |  | Shi470 (hspAmerind) |

|  |  | India7 (HpAsia2) |
| --- | --- | --- |
|  | P12 | ---- |
|  | Lithuania75 | ---- |
|  | G27 | ---- |
|  | HPAG1 | ---- |
| **hspEAsia** | DU15 | ---- |
|  | F28 | F63 F51 F78 F209 F210 F211 F24 F55 F75 MKF3 MKM1 MKF8 51 F16 F57 OK310 OK113  BM013A (HpSahul)  BM013B (HpSahul) |
|  | F20 | F21 F23 F94  F32 |
|  | F72 | F67  F90 |

|  |  | MKM6 |
| --- | --- | --- |
|  | F18 | MKF10 |
|  | ML1 | ML2  ML3 |
|  | 26695-1CH | 26695-1CL |
|  | UM298 | UM299 |
|  | oki102 | oki112 |
|  | G272 | ---- |
|  | MKM5 | ---- |
|  | 52 | ---- |
|  | F38 | ---- |
|  | F13 | ---- |
|  | F17 | ---- |
|  | F70 | ---- |
|  | F30 | ---- |
|  | NY40 | ---- |
|  | UM032 | ---- |
|  | UM037 | ---- |
|  | UM066 | ---- |
|  | oki128 | ---- |
|  | oki154 | ---- |
|  | oki422 | ---- |
|  | oki673 | ---- |
|  | oki828 | ---- |
|  | oki898 | ---- |
| **hspAmerind** | 7.13_R1a | 7.13_R2b  7.13_R1c  7.13_R3a |
|  | Cuz20 | Shi417 |
|  | 29CaP | ---- |

|  | 7C | ---- |
| --- | --- | --- |
|  | 26695-1 | ---- |
|  | Aklavik86 | ---- |
|  | Aklavik117 | ---- |
|  | ELS37 | ---- |
|  | SJM180 | ---- |
|  | Sat464 | ---- |
|  | Shi112 | ---- |
|  | Shi169 | ---- |
| **HpAsia2** | L7 | K26A1 (hspSAfrica) |
|  | SNT49 | PeCan4 (hspAmerind)  PeCan18 (hspAmerind) |
| **hspSAfrica** | CC33C | Hp238 (hspEAsia) |
| **hspWAfrica** | J99 | ---- |
|  | Gambia94/24 | J166 (HpEurope) |
|  | 908 | ---- |
|  | 2017 | ---- |
|  | 2018 | ---- |
| **HpAfrica2** | SouthAfrica7 | ---- |
|  | SouthAfrica20 | Puno120 (hspAmerind)  Puno135 (hspAmerind) v225d (hspAmerind) |
| **HpSahul** | BM012A | ---- |
|  | BM012B | ---- |
|  | BM012S | ---- |
|  | ausabrJ05 | ---- |
|  | PNG84A | ---- |
|  | PMSS1 | ---- |
|  | SS1 | ---- |
