# Supplementary material for: Helicobacter pylori virulence factors: relationship between genetic variability and phylogeographic origin
Source: PeerJ. 2021 Nov 26;9:e12272. doi: 10.7717/peerj.12272 (PMC8628625; doi:10.7717/peerj.12272)
Supplement: Supplemental Information 7 — The ancestral orders A60 to A117 are shown in the phylogeny of figure S2. Adhesins are represented from 1 to 10 and their orientation in the genome is represented by plus/plus (+) or plus/minus (−). [file peerj-09-12272-s007.docx]

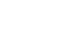

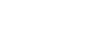
Supplementary Table S5**.** Ancestral rearrangement of adhesins compared to the reference *Helicobacter pylori* (strain 26695). The ancestral orders A60 to A117 are shown in the phylogeny of Figure S2. The adhesins are represented from 1 to 10 and their orientation in the genome is represented by plus/plus (+) or plus/minus (-).


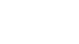

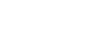


| **ANCESTRAL ARRANGEMENT OF ADHESINS** | | | |
| --- | --- | --- | --- |
| 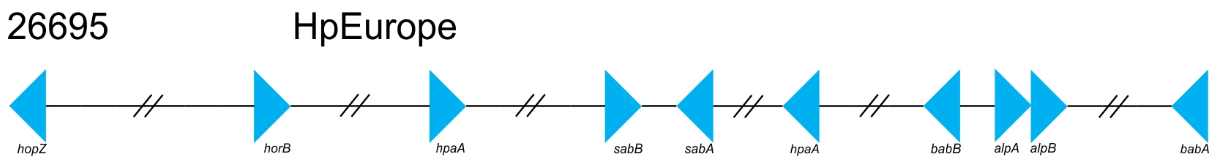  **-1**  **2**  **3**  **4**  **-5**  **-6**  **-7**  **-10**  **8 9** | | | |
| ***A60*** | -1 2 3 4 -5 -6 -7 8 9 -10 | ***A88*** | -1 2 -3 4 -5 -6 -10 8 9 -7 |
| ***A61*** | -1 7 2 -10 -9 -8 -3 4 -5 -6 | ***A89*** | -1 2 -3 -4 -5 -6 -10 8 9 -7 |
| ***A62*** | -1 2 3 -4 -5 -6 -9 -8 10 -7 | ***A91*** |  |
| ***A63*** | -1 2 3 4 5 6 -10 8 9 -7 | ***A90*** | -1 2 -3 -9 -8 10 6 5 4 -7 |
| ***A64*** |  | ***A92*** | -1 2 3 4 -5 6 -10 8 9 -7 |
| ***A65*** |  | ***A94*** |  |
| ***A68*** |  | ***A93*** | -1 2 3 -9 -8 7 6 5 -4 -10 |
| ***A69*** |  | ***A95*** | 1 2 3 4 5 6 -10 8 9 -7 |
| ***A71*** |  | ***A96*** | -1 2 -3 4 -5 -6 -10 8 9 -7 |
| ***A66*** | 1 2 3 4 5 6 -10 8 9 -7 | ***A97*** | -1 2 -3 -4 -5 -6 -10 8 9 -7 |
| ***A67*** | -1 -2 3 4 5 6 -10 8 9 -7 | ***A98*** | -1 2 -5 -4 -3 -10 8 9 -6 -7 |
| ***A70*** | -1 2 3 4 5 6 7 -9 -8 10 | ***A99*** | 7 -9 -8 10 -6 -5 -4 -3 -2 -1 |
| ***A72*** | -1 2 3 4 5 -10 8 9 -6 -7 | ***A100*** | 1 2 -5 -4 -3 -10 8 9 -6 -7 |
| ***A73*** |  | ***A101*** | -1 2 -3 -4 -5 -6 -10 7 8 9 |
| ***A74*** |  | ***A102*** | -1 2 -3 -4 -5 -6 7 8 9 -10 |
| ***A75*** | -1 2 3 4 5 6 -9 -8 10 -7 | ***A103*** | -1 2 3 4 5 6 7 8 9 -10 |
| ***A76*** | -1 2 3 4 5 6 -9 -8 7 -10 | ***A104*** | 1 2 -5 -4 -3 -10 8 9 -6 -7 |
| ***A78*** |  | ***A105*** | -1 -2 3 4 5 -10 8 9 -6 -7 |
| ***A77*** | -1 2 3 4 5 6 -7 8 9 -10 | ***A106*** | -1 -2 -3 -4 -5 -6 -10 8 9 -7 |
| ***A79*** |  | ***A107*** | -1 -2 -7 -9 -8 10 6 5 4 3 |
| ***A80*** |  | ***A108*** | 10 -9 -8 -7 -6 -5 -4 -3 -2 1 |
| ***A81*** | -1 2 3 4 5 6 7 8 9 10 | ***A109*** | -1 7 2 -10 8 9 -3 -4 -5 -6 |
| ***A82*** | -1 -2 3 -9 -8 10 -6 -5 -4 -7 | ***A110*** | -1 7 2 -10 -9 -8 -3 4 -5 -6 |
| ***A83*** | -1 2 3 4 -5 -6 -7 8 9 -10 | ***A111*** | -1 7 2 -10 8 9 -3 4 -5 -6 |
| ***A84*** |  | ***A112*** | -1 -2 -3 -4 -5 -6 -10 8 9 7 |
| ***A85*** | -1 2 3 4 -5 -6 -10 8 9 -7 | ***A113*** | -1 2 3 -10 4 -5 -6 -7 8 9 |
| ***A87*** |  | ***A114*** | -2 1 3 -10 -4 -5 -6 -7 8 9 |
| ***A86*** | -1 2 3 -4 -5 -6 -10 8 9 -7 | ***A115*** | -2 -4 -5 -6 -7 8 9 10 -3 -1 |
|  |  | ***A117*** | 2 -5 -4 -3 -10 8 9 -6 -7 1 |
